# Supplementary material for: SyNDI: synchronous network data integration framework
Source: BMC Bioinformatics. 2018 Nov 6;19:403. doi: 10.1186/s12859-018-2426-5 (PMC6219086; doi:10.1186/s12859-018-2426-5)
Supplement: Supplementary file 5 — Files of the example presented in Additional file 4. This additional file is a zip package that contains all files used in Additional file 4. Detailed descriptions of each of these files are presented in a README file included in this zip package. (GZ 30411 kb) [file 12859_2018_2426_MOESM5_ESM.gz › SyNDITools/Meme2Fimo/meme_4.9.0_4.tar.gz!/meme_4.9.0/website/html/mhmm-links.in]

Meta-MEME v3.3


> ### Related web sites
>
> ---
>
> - MEME is the motif discovery
>   tool that builds the motif models that serve as input to Meta-MEME.
>   MEME can be used to find motifs in DNA or protein sequences.
>   
> - MAST is a database search
>   tool that uses multiple MEME motif models to search a protein or
>   DNA database. MAST differs from Meta-MEME in that MAST treats each
>   motif model independently, rather than combining the motif models
>   into a single meta-model.
>   
> - SAM is a
>   hidden Markov model toolkit, developed at the University of
>   California, Santa Cruz. Unlike Meta-MEME, SAM models are not
>   motif-based. However, SAM supports both DNA and protein
>   models.
>   
> - HMMER is another hidden
>   Markov model toolkit. Developed by Sean Eddy at Washington
>   University in St. Louis, the HMMER toolkit includes a number of
>   homology detection programs for specific tasks. These can be used
>   with Meta-MEME by converting linear Meta-MEME models into HMMER
>   version 1.8 models.
>   
> - The BLOCKS database
>   is a searchable collection of PROSITE motifs. The associated
>   BLOCKMAKER
>   software allows for the construction of new motif blocks.
>   
> - Pfam is a database of
>   protein domain multiple alignments and HMMs.
>
> ---
>
> Return to the Meta-MEME home page.
>
> *Please send comments and questions to:
> @METAMEME\_CONTACT@.*
